# Congenital Anatomical Variations of the Posterior Cord of the Brachial Plexus in Human Fetuses: Branching Patterns and Surgical Implications

**DOI:** 10.7759/cureus.113855

**Published:** 2026-08-03

**Authors:** Lakshmi Kiruba N., Surekha Waghanna, Swapna B Parate, Dnyandeo Chopade, Amit Naik, Kumar Satish Ravi, Akhileshwar V Reddy

**Affiliations:** 1 Anatomy, SMBT Institute of Medical Sciences and Research Centre, Nashik, IND; 2 Anatomy, National Institute of Medical Sciences and Research, Jaipur, Jaipur, IND; 3 Obstetrics and Gynaecology, SMBT Institute of Medical Sciences and Research Centre, Nashik, IND; 4 Community Medicine, Dr. B.S. Tomar Institute of Medical Sciences and Research, Jaipur, IND

**Keywords:** anatomical variation, brachial plexus, fetal dissection, human fetus, posterior cord, regional anesthesia, surgical anatomy

## Abstract

Background: The posterior cord of the brachial plexus, formed by the posterior divisions of the upper, middle, and lower trunks (C5-T1), gives rise to the upper subscapular, thoracodorsal, lower subscapular, and axillary nerves and continues as the radial nerve. Variations in its formation and branching are clinically important for regional anesthesia (infraclavicular and axillary blocks), axillary surgery, and nerve-transfer procedures in brachial plexus palsy. Fetal dissection can reveal congenital patterns before postnatal or post-mortem distortion.

Objective: The single combined objective was split into two discrete, explicitly stated aims: (i) to document the prevalence and morphological types of variation in posterior cord formation and branching; and (ii) to test associations of these variations with gestational age, fetal sex, and side.

Materials and methods: Twenty-three formalin-preserved aborted human fetuses (gestational age 20-40 weeks; 46 upper limbs) were dissected bilaterally in the Department of Anatomy, SMBT Institute of Medical Sciences and Research Centre, Nashik, India. The pectoral, axillary, and arm regions were exposed to trace the roots, trunks, divisions, posterior cord, and its branches. Variations were classified by root contribution, posterior cord formation, and branching pattern. Associations with gestational age, fetal sex, and side were tested using the chi-square and Fisher’s exact tests (IBM SPSS Statistics for Windows, Version 25 (Released 2017; IBM Corp., Armonk, New York, United States); P<0.05 considered significant); odds ratios (ORs) with 95% confidence intervals (CIs) were calculated where appropriate.

Results: Posterior cord variations were present in 28 of 46 limbs (60.87%). The classical three-posterior-division pattern was seen in 37 limbs (80.43%), whereas variant formations (one, two, three (united distally), or four posterior divisions) occurred in nine limbs (19.57%). Prefixed roots (C4-T1 or C3-T1) were present in six limbs (13.04%). Branching was classical in 21 limbs (45.65%); the commonest anomaly was a common trunk for the axillary and lower subscapular nerves (10 limbs, 21.74%). Variations were significantly more frequent on the right side (18/23, 78.26%) than the left (10/23, 43.48%; χ^2^=5.84, P=0.016; OR 4.68, 95% CI 1.29-16.98) but were not associated with gestational age or fetal sex.

Conclusions: The posterior cord shows substantial congenital variability in human fetuses, with a right-sided predominance. Awareness of these patterns may inform surgical planning, neurotization, and regional anesthesia, with the potential to reduce the risks of incomplete blockade and iatrogenic nerve injury.

## Introduction

The brachial plexus is a complex neural network formed by the ventral rami of the fifth cervical to the first thoracic spinal nerves (C5-T1) that supplies motor, sensory, and sympathetic fibers to the upper limb. Among its components, the posterior cord is of particular clinical importance because of its terminal branches - the upper subscapular, thoracodorsal, lower subscapular, and axillary nerves, together with its continuation as the radial nerve - and its intimate relationship with the axillary vessels, which makes its variations highly relevant to surgical and anesthetic procedures [1-4].

Although the classical anatomy is well described, the brachial plexus shows considerable morphological variation arising from its embryological development and vascular relationships. Such variation may alter branching patterns and is frequently encountered in fetal studies, underscoring the developmental basis of adult plexus anatomy [2,5].

These variations have important clinical implications during neck and axillary surgery, regional anesthesia, trauma management, and the interpretation of nerve palsies. They are also relevant to obstetric brachial plexus injury, which occurs in approximately 0.5-3 per 1,000 live births and may cause permanent neurological deficits [6].

Fetal dissection reveals the primary embryologic pattern with minimal postnatal or post-mortem distortion. Against this background, the present study was undertaken with two specific aims: first, to document the prevalence and morphological types of variation in the formation and branching pattern of the posterior cord of the brachial plexus in human fetuses; and second, to determine whether these variations are associated with gestational age, fetal sex, and side. Together, these aims provide baseline morphological data of surgical and clinical relevance in an Indian fetal population.

## Materials and methods

Study design and setting

This observational morphological study was conducted in the Department of Anatomy, SMBT Institute of Medical Sciences and Research Centre (SMBTIMSRC), Nashik, Maharashtra, India. Bilateral dissections were performed on 23 formalin-preserved aborted human fetuses, yielding 46 upper limbs for analysis.

Sample characteristics

The 46 limbs comprised 28 male (60.87%; from 14 fetuses) and 18 female (39.13%; from nine fetuses) specimens, equally distributed between the right (n=23, 50%) and left (n=23, 50%) sides. Gestational age ranged from 20 to 40 weeks and was stratified into five groups: 20 to <24 weeks (n=8, 17.39%), 24 to <28 weeks (n=6, 13.04%), 28 to <32 weeks (n=16, 34.78%), 32 to <36 weeks (n=6, 13.04%), and 36 to <40 weeks (n=10, 21.74%).

Inclusion and exclusion criteria

Fetuses with a gestational age of at least 20 weeks and no gross congenital malformation of the upper limbs or neck were included. Specimens showing postmortem autolysis or visible deformity of the upper limb were excluded.

Dissection procedure

Standard anatomical dissection was performed using the posterior triangle approach with conventional dissection instruments (scalpel, fine dissecting forceps, and blunt probes). Skin, superficial fascia, and deep connective tissue were removed sequentially from the pectoral, axillary, and proximal arm regions to expose the brachial plexus. The roots (C5-T1), trunks, divisions, posterior cord, and terminal branches (upper subscapular, thoracodorsal, lower subscapular, and axillary nerves, and the radial nerve) were then traced systematically in a proximal-to-distal sequence - roots, followed by trunks, divisions, the posterior cord, and finally its terminal branches. The third part of the axillary artery was used as the principal anatomical landmark for defining the level and pattern of posterior cord formation. All structures and variations were photographed for the record and documented using standardized anatomical nomenclature [7].

Ethical approval

The study was approved by the Institutional Ethics Committee of SMBT Institute of Medical Sciences and Research Centre (approval number IEC-1401/SMBT/IMSRC/10/IEC/24/65, dated 02/02/2024). All formalin-preserved fetal specimens were obtained and handled in accordance with institutional and national ethical guidelines governing the use of human cadaveric material for anatomical research.

Data collection and classification

Variations were classified according to three parameters: (1) root contribution (normal C5-T1; prefixed C4-T1 or C3-T1); (2) posterior cord formation (normal three posterior divisions (3PD); and the variants one (1PD), two (2PD), variant three (3PD uniting distal to the third part of the axillary artery), or four (4PD) posterior divisions); and (3) branching pattern (classical anatomy versus common trunks and anomalous origins). Data were recorded on a predesigned proforma capturing fetal demographics and anatomical findings.

All specimens meeting the inclusion criteria during the study period were dissected consecutively, and none were selectively excluded, thereby limiting selection bias. To reduce observer bias, each limb was dissected and independently verified by two observers, and any discrepancy in interpretation was resolved by consensus with reference to the photographic record.

Statistical analysis

Categorical data were analyzed using the chi-square test or Fisher’s exact test, as appropriate. Odds ratios (ORs) with 95% confidence intervals (CIs) were calculated. Statistical significance was set at P<0.05. Analyses were performed using IBM SPSS Statistics for Windows, Version 25 (Released 2017; IBM Corp., Armonk, New York, United States).

## Results

A total of 46 upper limbs from 23 human fetuses were dissected. Posterior cord variations were observed in 28 limbs (60.87%), while 18 limbs (39.13%) showed classical morphology. The distribution of study variables is summarized in Table 1.

**Table 1:** Distribution of study variables (n=46; fetal upper limbs). *Variant 3PD: the posterior cord was formed distal to (posterior to) the third part of the axillary artery. AN: axillary nerve; LSN: lower subscapular nerve; PD: posterior division; SSN: subscapular nerve; TDN: thoracodorsal nerve; USN: upper subscapular nerve; 1PD/2PD/3PD/4PD: posterior cord formed by one/two/three/four posterior divisions

Variable	Category	n	%
Gestational age (weeks)	20 to <24	8	17.39
	24 to <28	6	13.04
	28 to <32	16	34.78
	32 to <36	6	13.04
	36 to <40	10	21.74
Fetal sex	Male	28	60.87
	Female	18	39.13
Side	Right	23	50.00
	Left	23	50.00
Root pattern	Normal (C5-T1)	40	86.96
	Prefixed (C4-T1)	4	8.70
	Prefixed (C3-T1)	2	4.35
Posterior cord formation	Classical (3PD)	37	80.43
	Single posterior division (1PD)	1	2.17
	Two posterior divisions (2PD)	4	8.70
	Variant 3PD (united distally)*	3	6.52
	Four posterior divisions (4PD)	1	2.17
Posterior cord branching	Classical	21	45.65
	Common trunk: AN + LSN	10	21.74
	AN + USN from PD of upper trunk	4	8.70
	USN from PD of upper trunk	4	8.70
	Common trunk: TDN + LSN, with three SSN	3	6.52
	Common trunk: AN + LSN, with three SSN	2	4.35
	Common trunk: AN + TDN + LSN	1	2.17
	Common trunk: TDN + LSN	1	2.17
Overall	Classical	18	39.13
	Variation	28	60.87
Total		46	100.00

Posterior cord branching patterns

**Figure 1:**
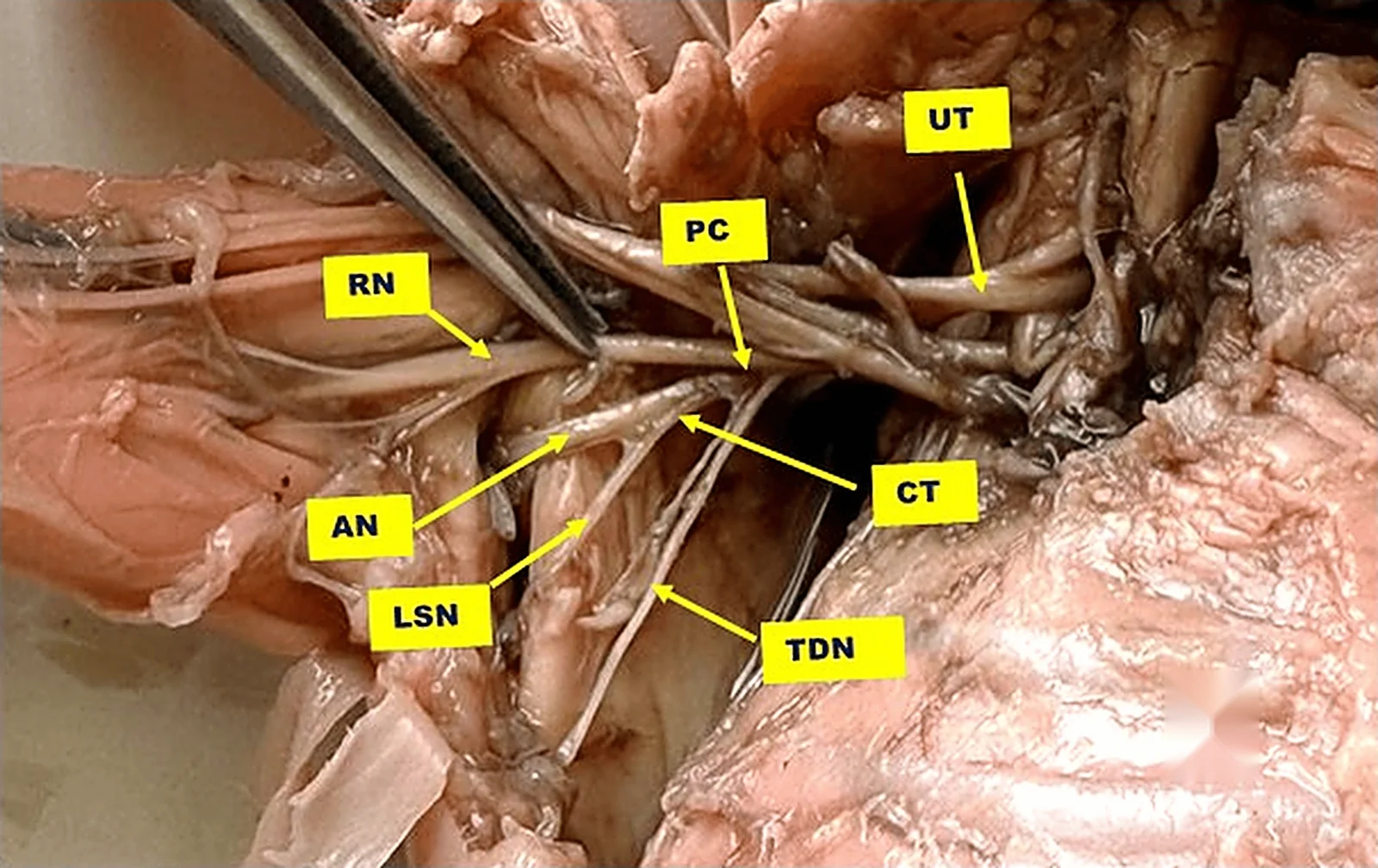
Common trunk of origin for the axillary and lower subscapular nerves. Fetal axilla: the axillary nerve (AN) and lower subscapular nerve (LSN) arise from a single common trunk (CT) of the posterior cord. TDN: thoracodorsal nerve; UT: upper trunk; PC: posterior cord; RN: radial nerve

**Figure 2:**
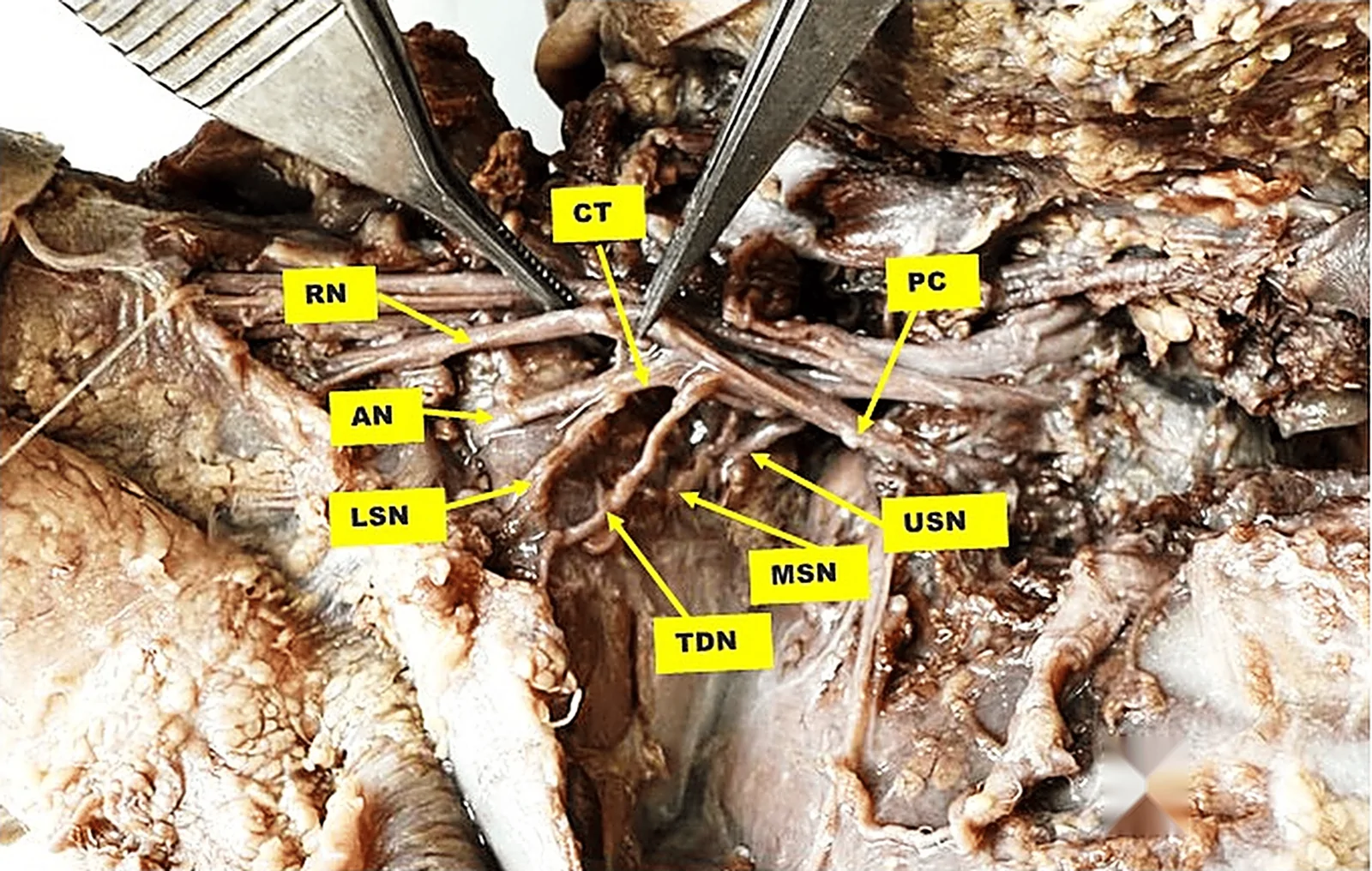
Common trunk for the axillary and lower subscapular nerves with three subscapular nerves. A common AN-LSN trunk is present together with three subscapular nerves arising from the posterior cord. AN: axillary nerve; LSN: lower subscapular nerve; TDN: thoracodorsal nerve; USN: upper subscapular nerve; MSN: middle subscapular nerve; PC: posterior cord; RN: radial nerve; CT: common trunk

**Figure 3:**
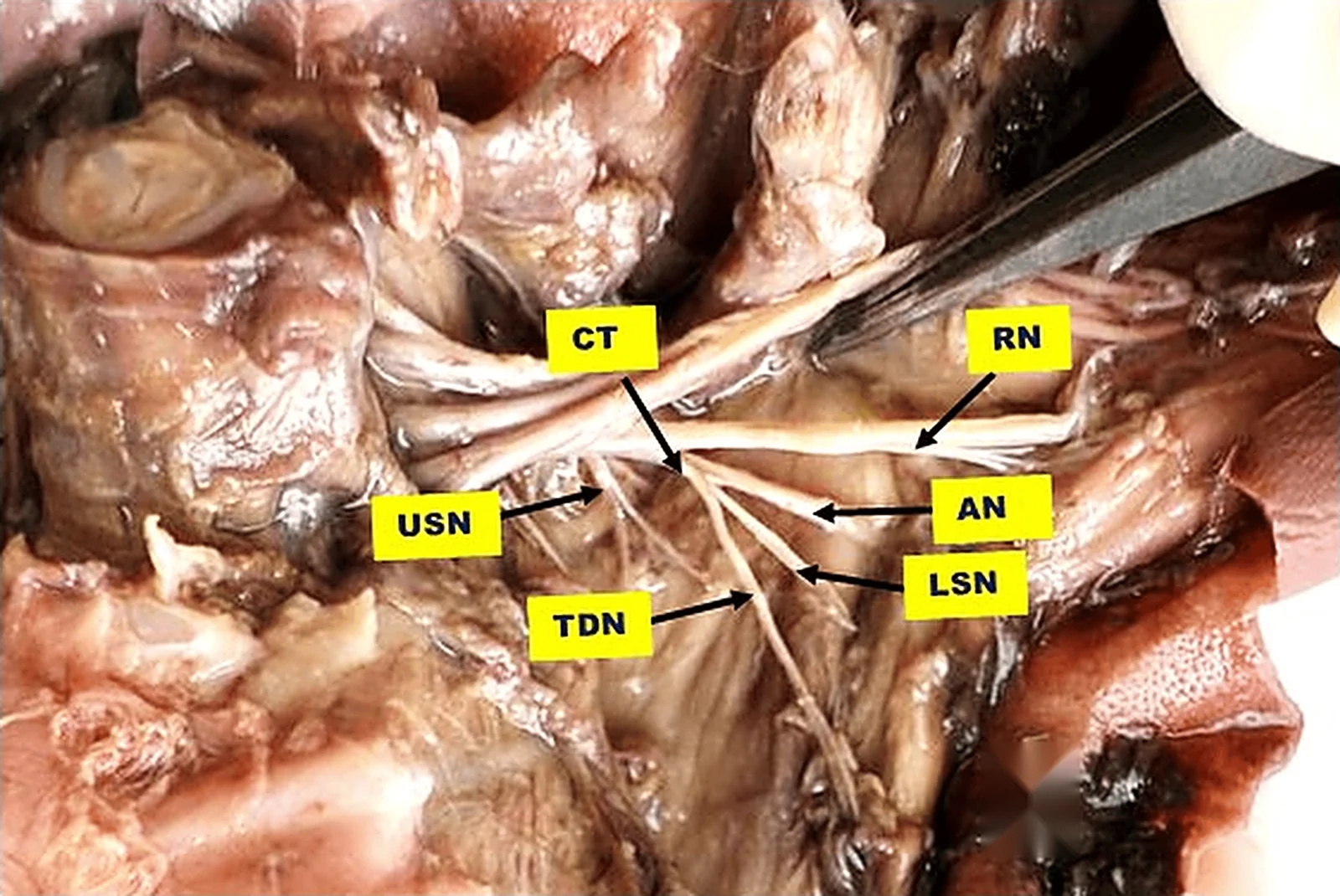
Common trunk of origin for the axillary, thoracodorsal, and lower subscapular nerves. The AN, TDN, and LSN share a single common trunk from the posterior cord. AN: axillary nerve; LSN: lower subscapular nerve; TDN: thoracodorsal nerve; USN: upper subscapular nerve; RN: radial nerve; CT: common trunk

**Figure 4:**
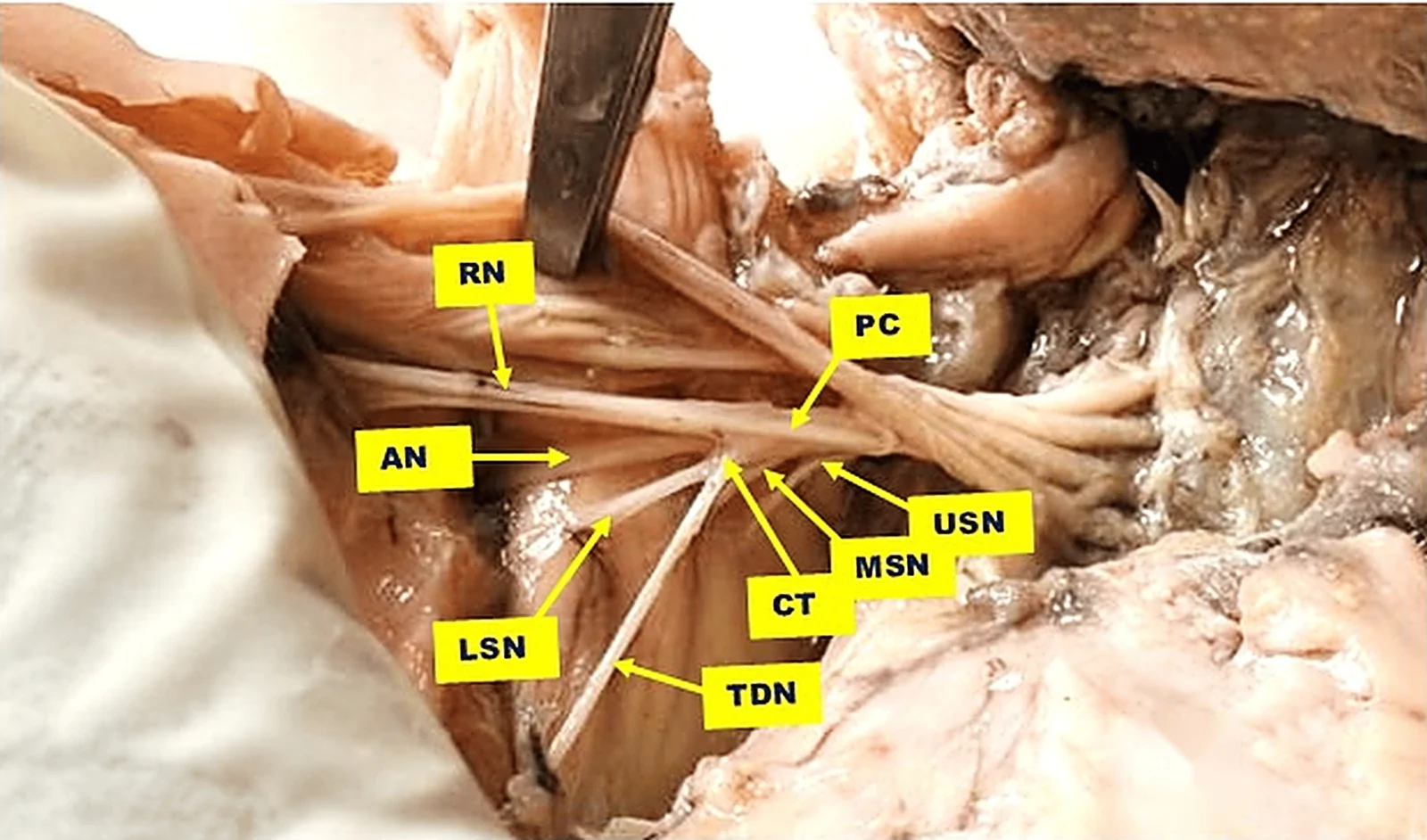
Common trunk for the thoracodorsal and lower subscapular nerves with three subscapular nerves. A common TDN-LSN trunk is associated with three subscapular nerves. AN: axillary nerve; LSN: lower subscapular nerve; TDN: thoracodorsal nerve; USN: upper subscapular nerve; MSN: middle subscapular nerve; PC: posterior cord; RN: radial nerve; CT: common trunk

**Figure 5:**
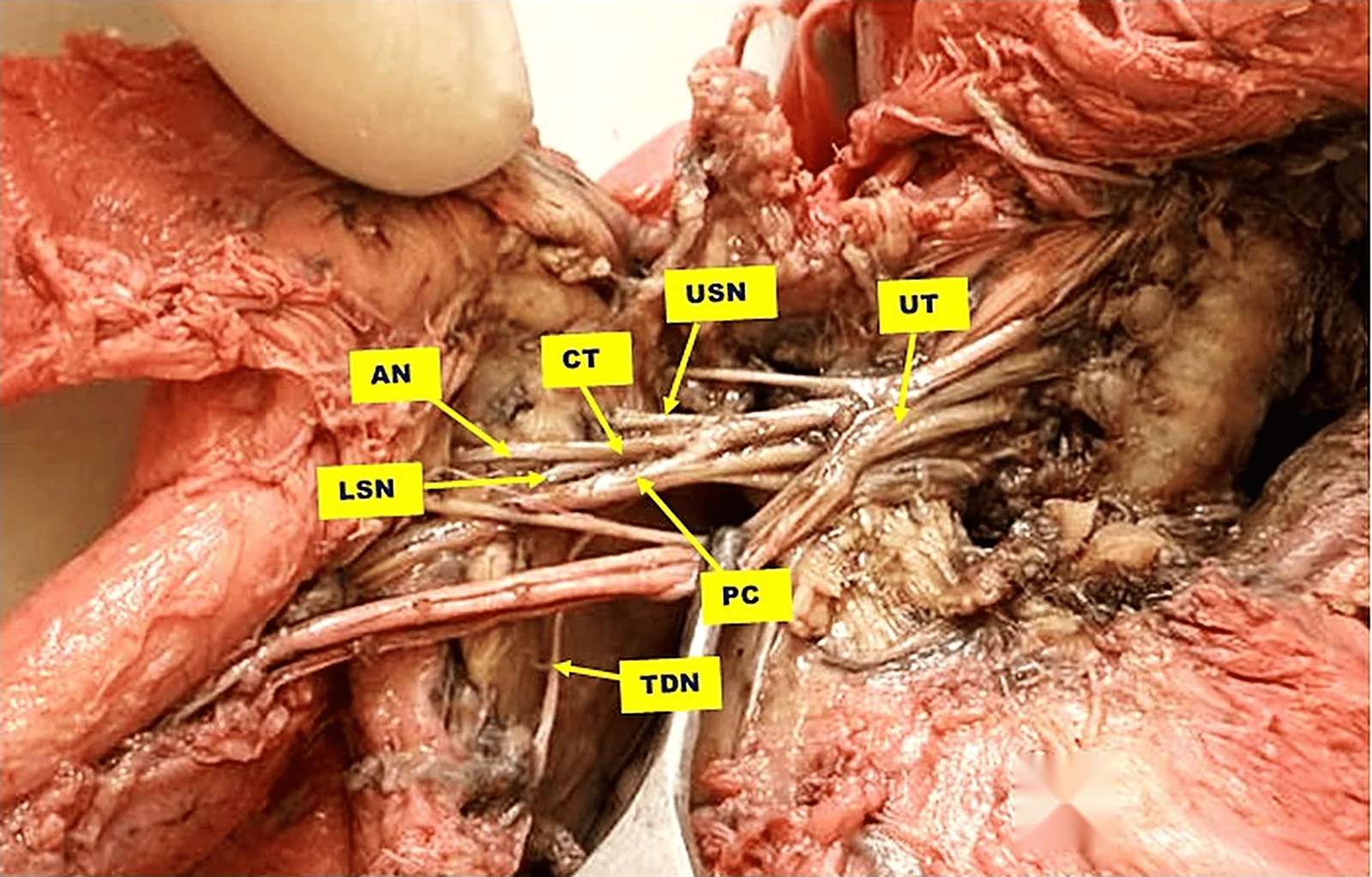
Axillary and upper subscapular nerves arising from the posterior division of the upper trunk. The AN and USN originate from the posterior division of the upper trunk rather than from the fully formed posterior cord. AN: axillary nerve; LSN: lower subscapular nerve; TDN: thoracodorsal nerve; USN: upper subscapular nerve; MSN: middle subscapular nerve; PC: posterior cord; CT: common trunk

**Figure 6:**
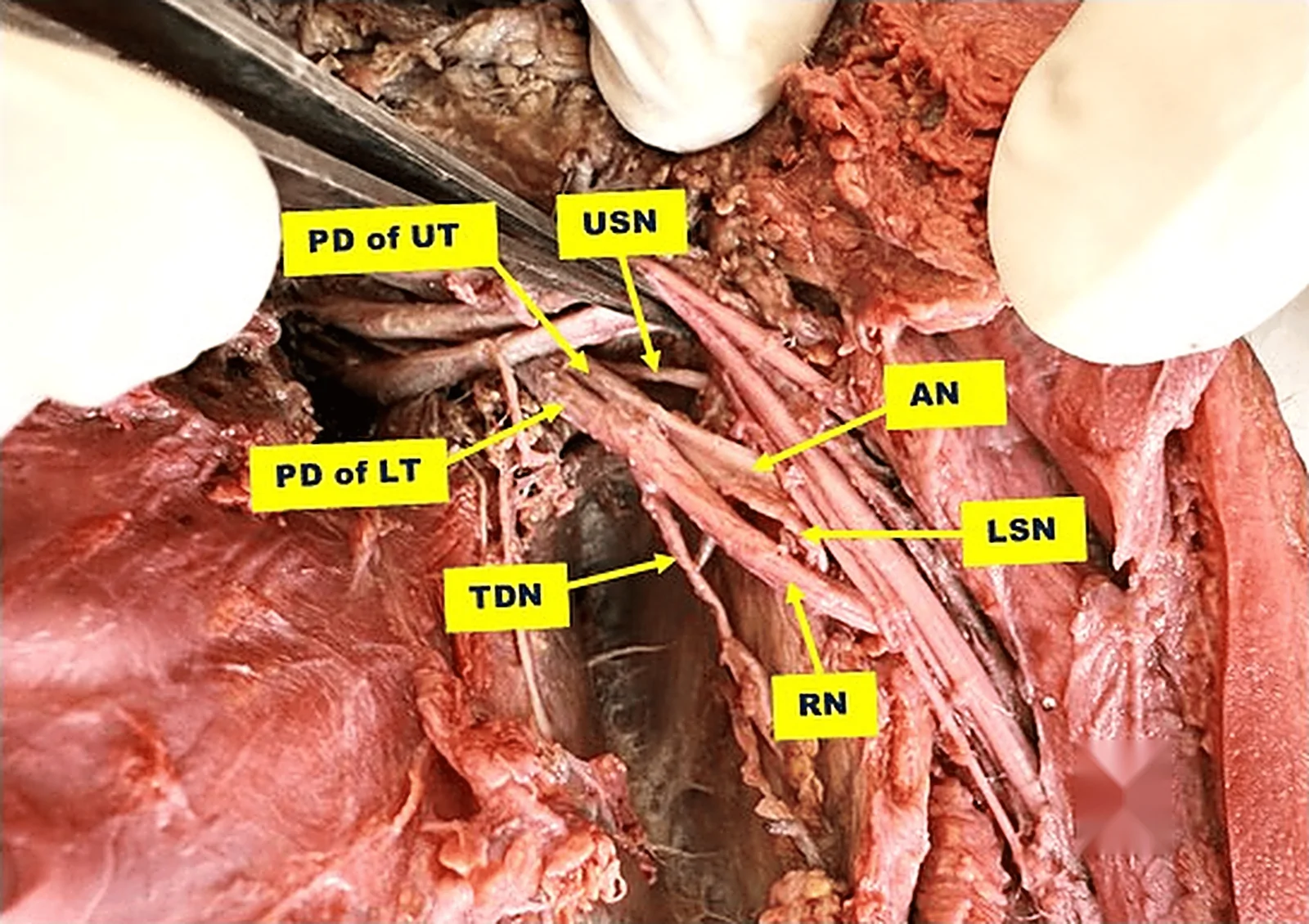
Upper subscapular nerve arising from the posterior division of the upper trunk. The USN takes an early origin from the posterior division of the upper trunk. AN: axillary nerve; LSN: lower subscapular nerve; TDN: thoracodorsal nerve; USN: upper subscapular nerve; MSN: middle subscapular nerve; PD: posterior division; RN: radial nerve

Classical branching of the posterior cord was observed in 21 limbs (45.65%). Among the variants, a common trunk of origin for the axillary and lower subscapular nerves was the commonest, occurring in 10 limbs (21.74%; Figure 1). A common trunk for the axillary and lower subscapular nerves, accompanied by three subscapular nerves, was seen in two limbs (4.35%; Figure 2), and a common trunk for the axillary, thoracodorsal, and lower subscapular nerves was seen in one limb (2.17%; Figure 3). A common trunk for the thoracodorsal and lower subscapular nerves occurred in one limb (2.17%), and the same pattern with three subscapular nerves in three limbs (6.52%; Figure 4). The axillary and upper subscapular nerves arose together from the posterior division of the upper trunk in four limbs (8.70%; Figure 5), and the upper subscapular nerve alone arose from the posterior division of the upper trunk in a further four limbs (8.70%; Figure 6). Overall, three subscapular nerves were present in five limbs (10.87%).

Root variations

**Figure 7:**
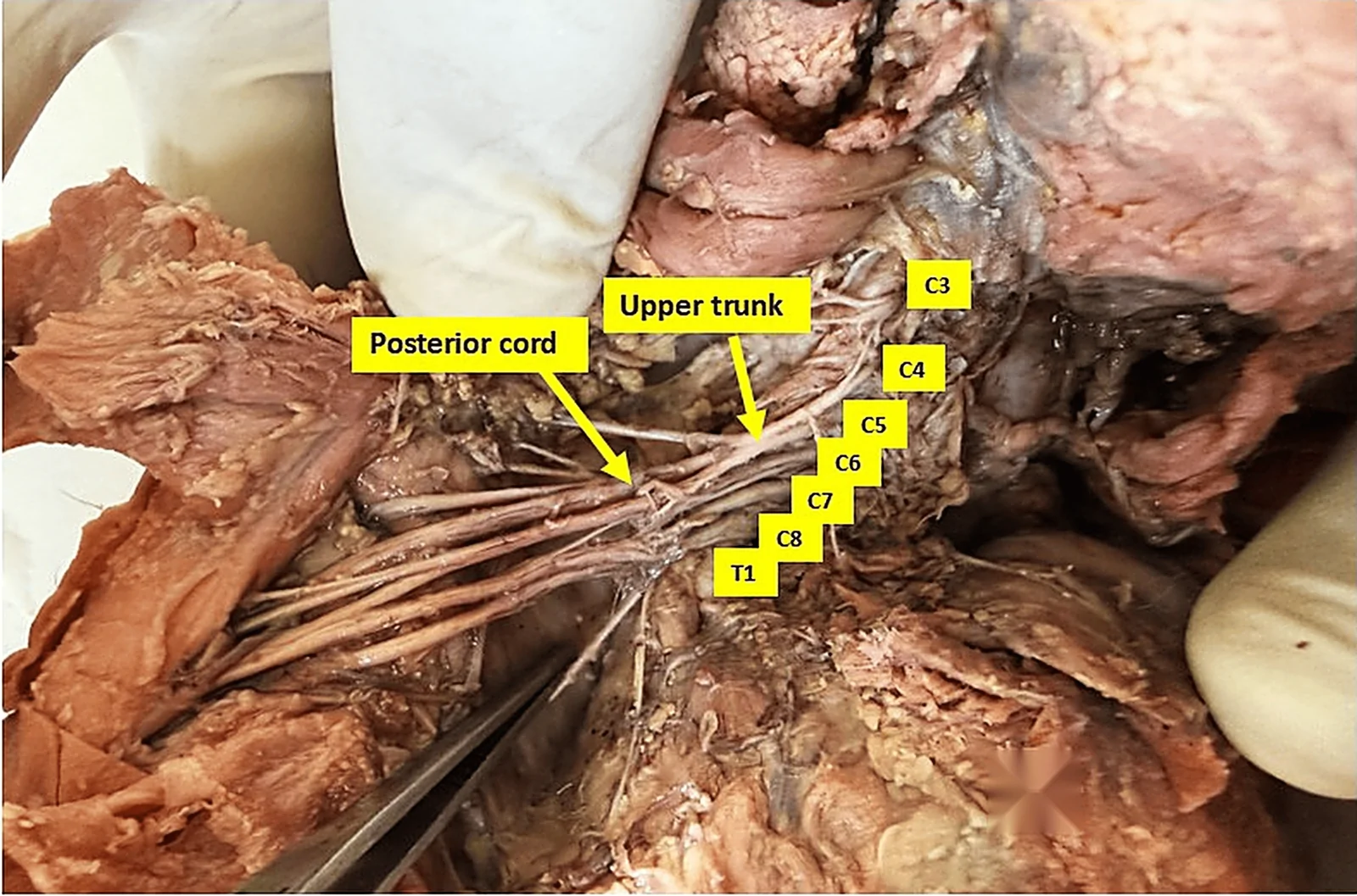
Prefixed roots of the brachial plexus (C3-T1). The plexus receives a prefixed contribution, with the C3 root joining the formation (C3-T1 pattern).

The roots followed the normal C5-T1 pattern in 40 limbs (86.96%). Prefixed contributions were present in six limbs (13.04%): a C4-T1 pattern in four limbs (8.70%) and a C3-T1 pattern in two limbs (4.35%; Figure 7). No postfixed plexus was identified.

Posterior cord formation

**Figure 8:**
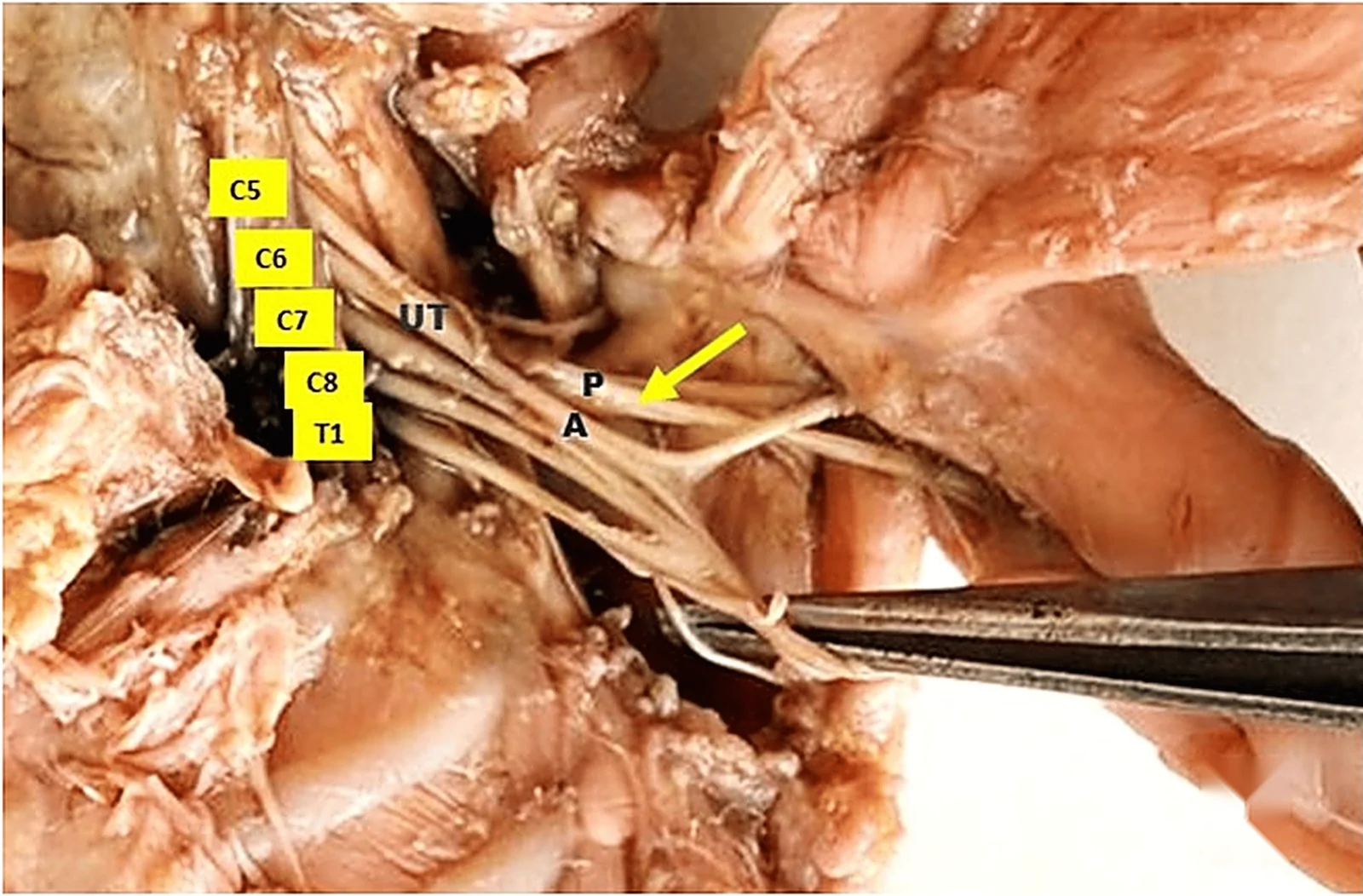
Posterior cord formed by a single posterior division (1PD) (arrow). PC: posterior cord

**Figure 9:**
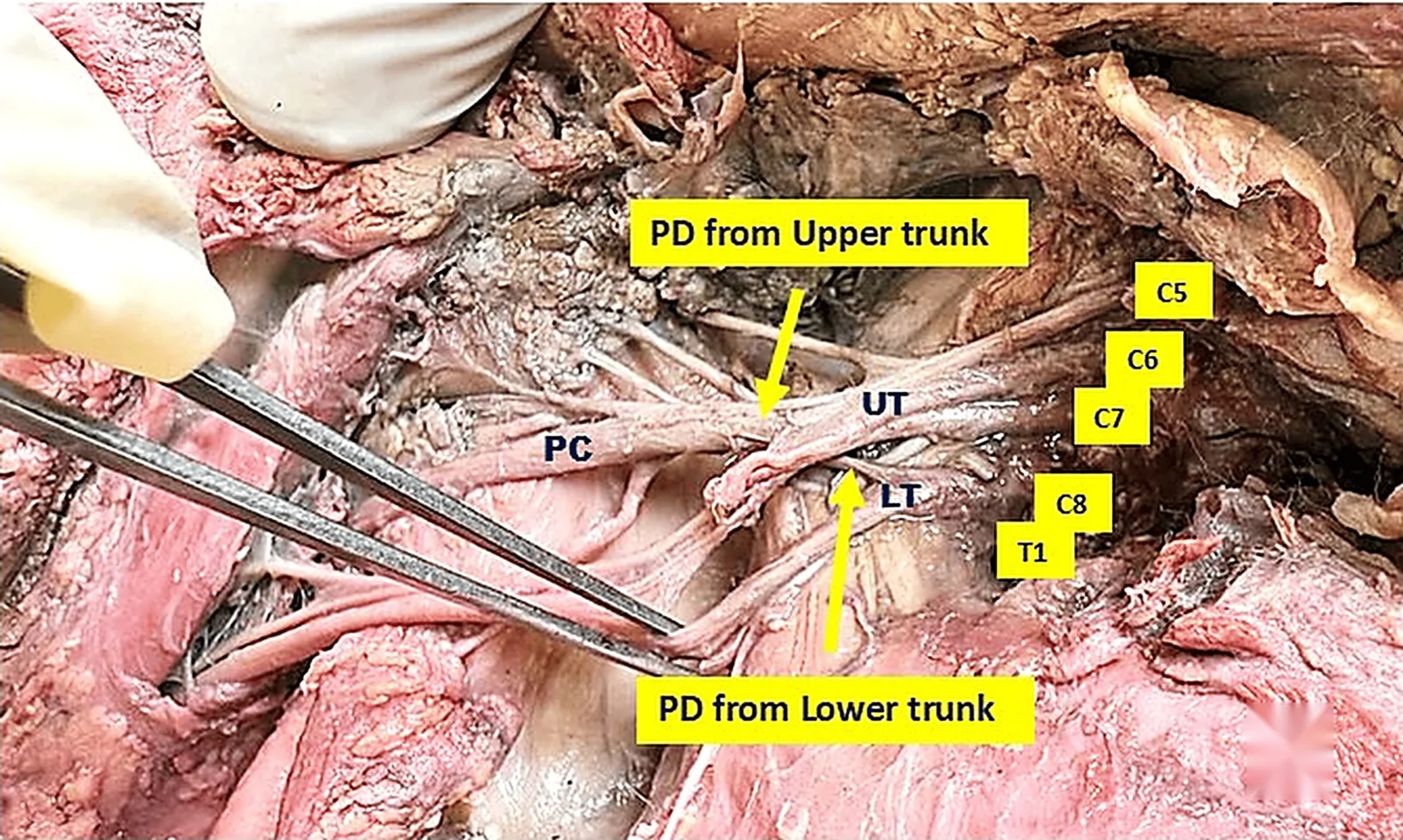
Posterior cord formed by two posterior divisions (2PD) (arrows). PC: posterior cord; UT: upper trunk; LT: lower trunk

**Figure 10:**
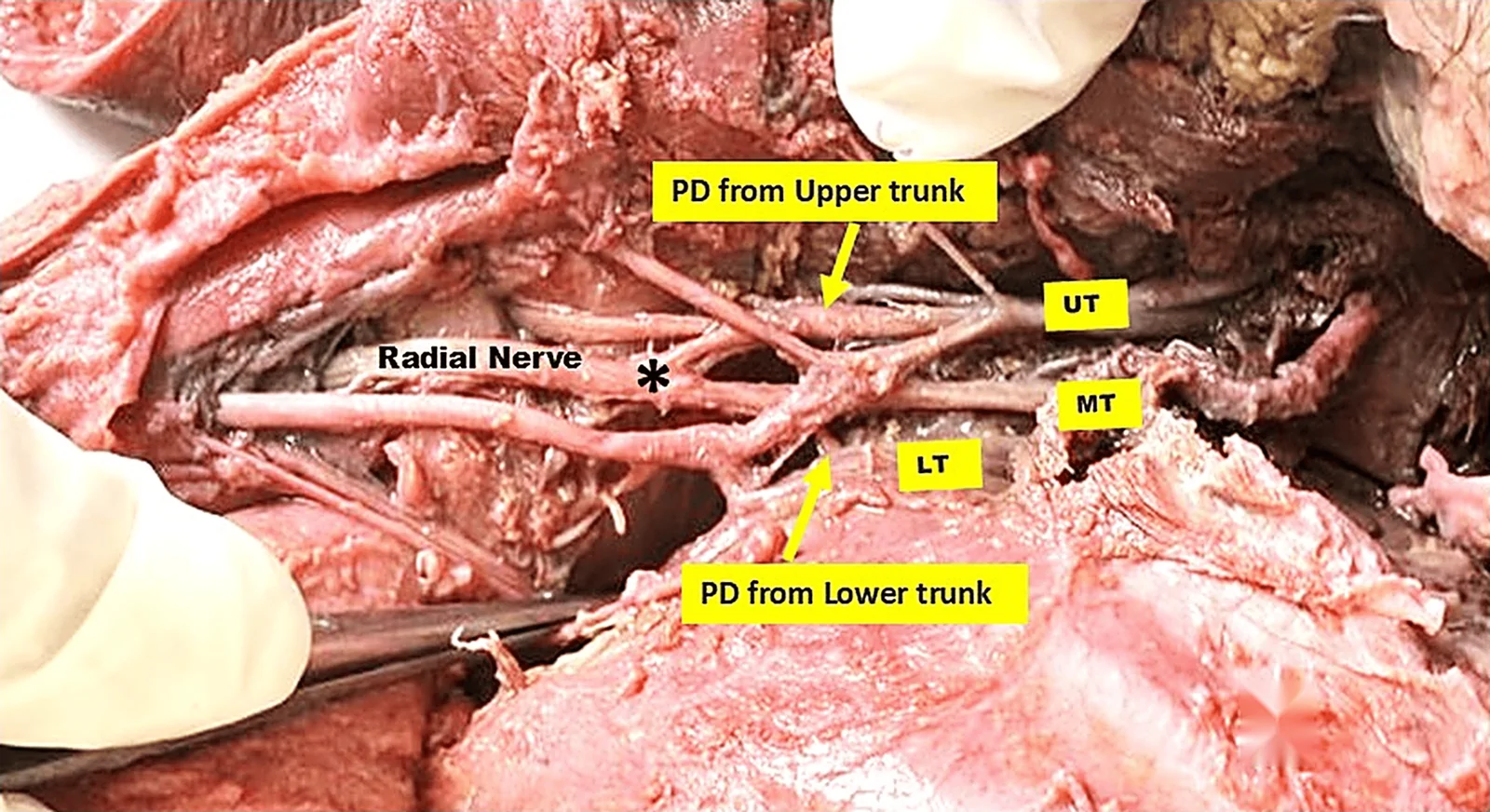
Posterior cord formed by three posterior divisions (3PDs) uniting posterior to the third part of the axillary artery (arrows). The 3PDs unite distal to the third part of the axillary artery. PC: posterior cord; UT: upper trunk; LT: lower trunk; MT: middle trunk

**Figure 11:**
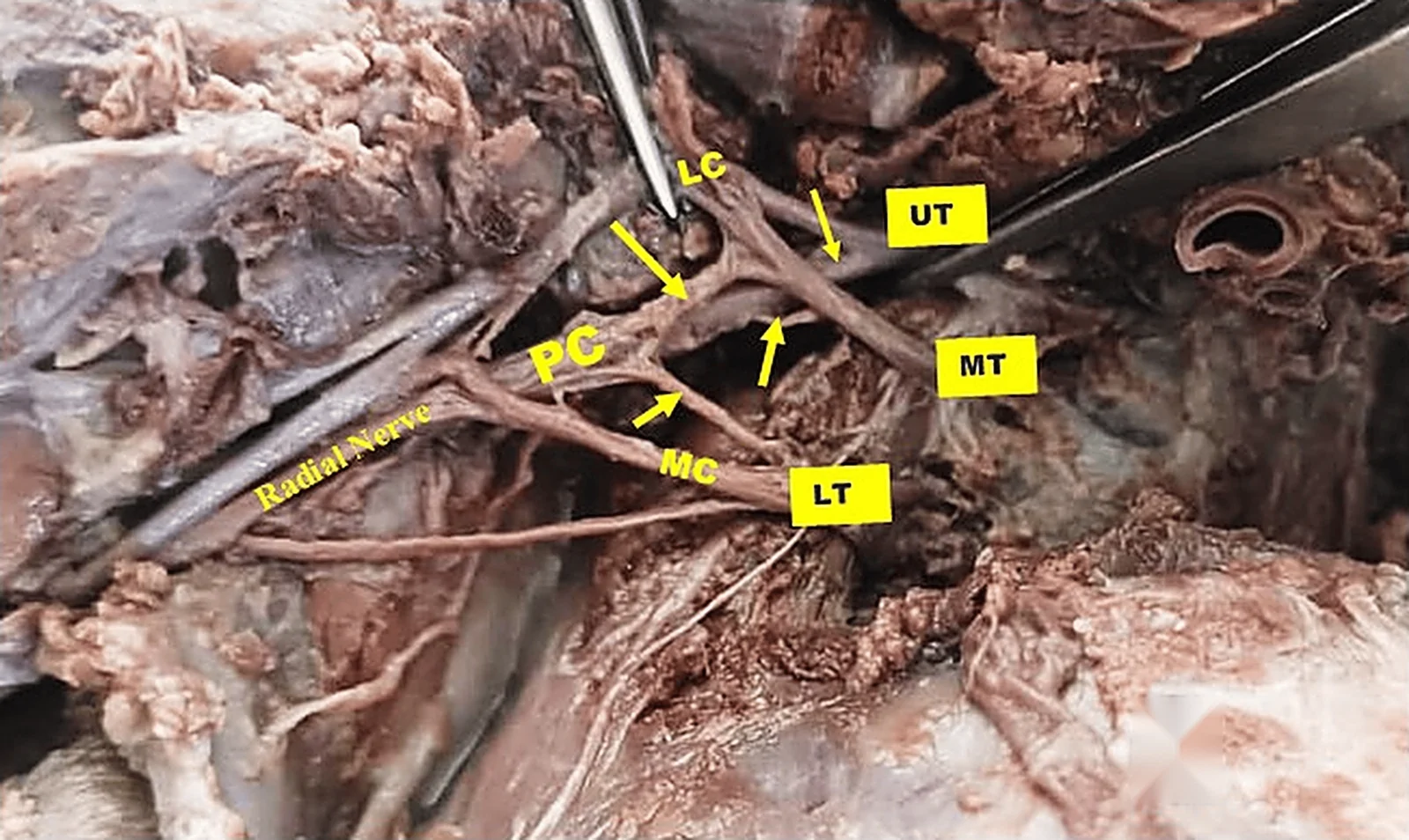
Posterior cord formed by four posterior divisions (4PD) (arrows). PC: posterior cord; MC: medial cord; LC: lateral cord; UT: upper trunk; LT: lower trunk; MT: middle trunk

The posterior cord was formed by the classical three posterior divisions in 37 limbs (80.43%). Variant formations were identified in nine limbs (19.57%). A single posterior division was seen in one limb (2.17%; Figure 8) and two posterior divisions in four limbs (8.70%; Figure 9). In three limbs (6.52%), the posterior cord was formed by three posterior divisions that united distal to the third part of the axillary artery (Figure 10), and four posterior divisions were seen in one limb (2.17%; Figure 11).

Associations with gestational age, sex, and side

The distribution of classical versus variant posterior cords across gestational age groups is shown in Table 2; no significant association was found (χ^2^=5.08, P=0.279). Fetal sex was likewise not significantly associated with variation (χ^2^=0.0007, P=0.979; Table 3). In contrast, variations were significantly more frequent on the right side than on the left (χ^2^=5.84, P=0.016; OR 4.68, 95% CI 1.29-16.98; Table 4).

**Table 2:** Association between gestational age group and posterior cord variation. Chi-square test: χ^2^=5.08, P=0.279 (not significant).

Gestational age (weeks)	Classical, n (%)	Variation, n (%)	Total, n (%)
20 to <24	1 (12.50)	7 (87.50)	8 (100)
24 to <28	2 (33.33)	4 (66.67)	6 (100)
28 to <32	9 (56.25)	7 (43.75)	16 (100)
32 to <36	3 (50.00)	3 (50.00)	6 (100)
36 to <40	3 (30.00)	7 (70.00)	10 (100)
Total	18 (39.13)	28 (60.87)	46 (100)

**Table 3:** Association between fetal sex and posterior cord variation. Chi-square test: χ^2^=0.0007, P=0.979 (not significant).

Fetal sex	Classical, n (%)	Variation, n (%)	Total, n (%)
Female	7 (38.89)	11 (61.11)	18 (100)
Male	11 (39.29)	17 (60.71)	28 (100)
Total	18 (39.13)	28 (60.87)	46 (100)

**Table 4:** Association between side and posterior cord variation. Chi-square test: χ^2^=5.84, P=0.016 (significant). Odds ratio (right vs. left)=4.68, 95% CI 1.29-16.98.

Side	Classical, n (%)	Variation, n (%)	Total, n (%)
Left	13 (56.52)	10 (43.48)	23 (100)
Right	5 (21.74)	18 (78.26)	23 (100)
Total	18 (39.13)	28 (60.87)	46 (100)

## Discussion

The present study demonstrates a high prevalence (60.87%) of posterior cord variation across 46 fetal upper limbs. This is consistent with earlier fetal series, which report overall brachial plexus variation rates of roughly 25-67% that arise during early embryogenesis rather than as postnatal adaptation [2,8,9]. Neither fetal sex nor gestational age was significantly associated with variation, but a statistically significant right-sided predominance (P=0.016) was observed, a finding with potential implications for surgical, diagnostic, and anesthetic procedures involving the brachial plexus.

Brachial plexus formation occurs during the fifth to seventh weeks of gestation, as the ventral rami grow into the limb bud under molecular guidance and the mechanical constraints imposed by the developing humerus and vasculature. The subclavian and axillary arterial precursors influence the pattern of divisions; atypical arterial relationships may disrupt the symmetric fusion of the posterior divisions, which could account for the multiple posterior-division configurations observed here (2PD, 8.70%; variant 3PD, 6.52%; 1PD and 4PD, 2.17% each). The prefixed root contributions (C4-T1 or C3-T1; 13.04% combined) are also in keeping with previous fetal reports [2,10].

Classical posterior cord formation occurred in 80.43% of limbs, comparable to adult cadaveric rates of approximately 70-90% [3]. The commonest branching anomaly - a common trunk for the axillary and lower subscapular nerves (21.74%) - mirrors adult observations in which the lower subscapular nerve frequently shares an origin with the axillary nerve, in up to 57% of limbs [4]. Origin of the upper subscapular nerve, or of the axillary and upper subscapular nerves together, from the posterior division of the upper trunk (17.4% combined) reflects early cord divergence and has been reported in comparable proportions in other series [3,4].

Three subscapular nerves were present in 5 of 46 limbs (10.87%), indicating segmentation variability within the subscapular nerve group. Such multiplicity may be asymptomatic but is surgically relevant, because inadvertent injury to small branches during axillary dissection can produce subtle weakness of medial rotation and adduction or alter scapulohumeral mechanics. Subscapular branches are also used for neurotization, most often to reinnervate the axillary nerve and restore deltoid function after birth-related injury [11].

These fetal variants are anatomically relevant to neonatal brachial plexus palsy (incidence approximately 1-3 per 1,000 births), in which altered cord biomechanics may plausibly influence the risk of traction injury [12]. Where such variants are present, surgeons performing axillary dissection, infraclavicular blocks, or neurotization (for example, using thoracodorsal or lower subscapular grafts) may benefit from anticipating common trunks in order to reduce the risk of iatrogenic damage, and the observed laterality bias suggests that heightened vigilance on the right side could be warranted. It should be emphasized, however, that the present study did not evaluate surgical outcomes, anesthetic success, or nerve-injury rates; these clinical implications therefore represent potential applications of the anatomical findings rather than effects directly demonstrated here [11,12].

The right-sided predominance reached statistical significance (OR 4.68, 95% CI 1.29-16.98); however, the width of the CI reflects the limited sample size, and this laterality finding should be regarded as preliminary until confirmed in larger cohorts.

Fetal dissection offers a unique window into the developmental determination of the brachial plexus pattern, revealing the primary embryologic template with minimal postnatal or postmortem distortion. Previous fetal studies have reported overall variation rates of 13-50%, with variable emphasis on posterior cord formation and branching [2,9,10]; the present series adds population-specific data with a detailed focus on the posterior cord.

Limitations

Several limitations should be acknowledged. First, the modest sample (23 fetuses; 46 limbs) drawn from a single institution and a single (Indian) region limits generalizability and precludes robust subgroup inference, particularly within individual gestational age strata. Second, because the specimens were fetal, extrapolation of these findings to adult anatomy should be made with caution. Third, as a cross-sectional observational study, the design permits description of anatomical variations and their associations but cannot establish causal or developmental-temporal relationships. Fourth, although observer bias was mitigated through independent dual assessment with consensus resolution, formal inter-observer agreement statistics were not computed. Larger, multicenter cohorts with imaging correlation are recommended to confirm these findings and to further define the developmental basis of posterior cord variation.

## Conclusions

The posterior cord of the brachial plexus exhibits substantial congenital variability in human fetuses, with a significant right-sided predominance and no association with fetal sex or gestational age. By documenting these patterns in an Indian fetal population, this study contributes population-specific data that enrich the global understanding of brachial plexus morphology, with particular relevance to obstetric and surgical practice. Awareness of posterior cord variations may be valuable for neurophysiological assessment and the diagnosis of peripheral nerve lesions, for planning nerve grafting and neurotization, and for safe regional anesthesia, with the potential to reduce the risks of incomplete blockade and iatrogenic injury. As these clinical benefits were not directly evaluated here, they are best regarded as potential applications that warrant confirmation in larger, imaging-correlated studies.
